# Brentuximab vedotin for skin involvement in refractory diffuse cutaneous systemic sclerosis, an open-label trial

**DOI:** 10.1093/rheumatology/keae235

**Published:** 2024-04-23

**Authors:** Andreu Fernández-Codina, Tatiana Nevskaya, Murray Baron, C Thomas Appleton, Matthew J Cecchini, Amanda Philip, Maha El-Shimy, Louise Vanderhoek, Iago Pinal-Fernández, Janet E Pope

**Affiliations:** Division of Rheumatology, Western University, London, ON, Canada; Division of General Internal Medicine-Windsor Campus, Western University, London, ON, Canada; Division of Systemic Autoimmune Diseases and Critical Care, Hospital Clinic de Barcelona, Barcelona, Spain; Division of Rheumatology, Western University, London, ON, Canada; Division of Rheumatology, Jewish General Hospital, McGill University, Montreal, QC, Canada; Division of Rheumatology, Western University, London, ON, Canada; Division of Pathology, Western University, London, ON, Canada; Division of Rheumatology, Western University, London, ON, Canada; Division of Rheumatology, Western University, London, ON, Canada; Division of Rheumatology, Western University, London, ON, Canada; Muscle Unit, National Institute of Arthritis and Musculoskeletal and Skin Diseases, Bethesda, MD, USA; Department of Neurology, Johns Hopkins University School of Medicine, Baltimore, MD, USA; Division of Rheumatology, Western University, London, ON, Canada

**Keywords:** Systemic sclerosis, treatment, biologic, skin, brentuximab

## Abstract

**Objective:**

We explored the efficacy and safety of brentuximab vedotin, a chimeric anti-CD30 antibody drug conjugate, in patients with severe active diffuse cutaneous systemic sclerosis (dcSSc).

**Methods:**

This phase II proof-of-concept, single centre, open-label, single arm, investigator-initiated trial included patients ≥18 years, with dcSSc, modified Rodnan skin score (mRSS) ≥15 with <5 years since the first non-Raynaud’s symptom and/or skin worsening despite immunosuppression who were treated with intravenous brentuximab vedotin 0.6 mg/kg q3 weeks for 45 weeks. The primary end point was a decrease in mRSS of ≥8 points at 48 weeks.

**Results:**

Eleven patients were treated with brentuximab vedotin, with nine completing the study. The mean mRSS reduction at week 48 was 11.3 (95% CI 6.9, 15.8; *P* = 0.001), meeting the primary end point in the intention to treat analysis (7/11 had a decrease in mRSS ≥8). The % forced vital capacity increased by 7.8% (12.5). The Composite Response Index in dcSSc (CRISS) suggested a beneficial treatment effect (86% ≥0.6). Most adverse events were mild. No SAEs were attributed to brentuximab vedotin.

**Conclusion:**

In dcSSc, brentuximab vedotin improved skin and FVC without safety concerns. A placebo-controlled trial is warranted to corroborate these initial findings.

**Trial registration:**

ClinicalTrials.gov, http://clinicaltrials.gov, NCT03198689.

Rheumatology key messages

- Brentuximab vedotin is a chimeric anti-CD30 antibody drug conjugate (ADC) approved to treat different lymphomas.
- Our study met its main endpoint with a reduction of the modified Rodnan skin score.
- We provide a new and safe potential therapeutic pathway in SSc.

## Introduction

Systemic sclerosis (SSc) is a rare autoimmune disease characterized by inflammation, fibrosis and vascular changes. The immune dysregulation in SSc is partially driven by a T lymphocyte response. Activated type 2 helper (Th2) lymphocytes, along with B lymphocytes and macrophages, produce different cytokines including transforming growth factor beta (TGF-B), interleukin 6 (IL-6) and interleukin 13 (IL-13) [1]. These factors, in combination with other substances coming from platelets and dendritic cells, lead to fibroblast activation and extracellular matrix overproduction followed by end-organ damage. Patients with a more severe skin involvement will have increased morbidity [2] and mortality [3]. To date, most treatments have shown, at most, modest benefit for scleroderma skin involvement (mycophenolate mofetil, methotrexate, cyclophosphamide, rituximab, tocilizumab). Only autologous hematopoietic stem cell transplantation (AHSCT) has demonstrated large improvement in the modified Rodnan skin score (mRSS) in highly selected patients [4, 5].

Th2 lymphocyte infiltration was demonstrated in diffuse cutaneous SSc (dcSSc) skin biopsies [6]. Patients had CD4+ cells in the skin expressing CD30 and elevated serum levels of free soluble CD30 (sCD30). CD30 is a member of the tumor necrosis factor-receptor superfamily. Brentuximab vedotin is an antibody-drug conjugate (ADC) against CD30, combining an IgG1 monoclonal antibody with the antimitotic agent monomethylauristatin E (MMAE). Once the CD30 receptor in the Th2 lymphocyte binds to the antibody, endocytosis releases MMAE, leading to cell cycle arrest and apoptosis. Brentuximab vedotin has been approved for advanced Hodgkin’s and non-Hodgkin’s lymphomas [7]. Although data were sparse, we wanted to determine whether brentuximab vedotin could improve the skin involvement of patients with severe disease and who in general had failed treatment.

We performed a phase 2 open-label, single-arm clinical trial as a proof-of-concept study to investigate the efficacy and safety of brentuximab vedotin for skin involvement in patients with severe active dcSSc.

## Methods

### Study design

This was a phase 2 open-label, single-arm, single-site clinical trial conducted at the Rheumatology Division, Western University, London, ON, Canada. We compared the results to matched historic controls from the Canadian Scleroderma Registry (CSRG). The study was conducted following the regulations from the Conference on Harmonization Guidelines for Good Clinical Practice and the Declaration of Helsinki. The study protocol was registered at Clinicaltrials.gov (NCT03198689). Institutional Review Board approval was from Western Health Sciences Ethics Research Board (project identification 111900). This was an investigator-initiated study. Seagen Inc. (now a wholly owned subsidiary of Pfizer Inc.) provided the study drug and some funding for the study. Seagen had no role in the final manuscript. The other funding was from J.E.P.’s research money at St Joseph’s Health Care, and Lawson Research Institute. Seagen, patients and public were not involved in the design of this trial.

### Participants

Adults over 18 years old, meeting the American College of Rheumatology/European League Against Rheumatism 2013 SSc classification criteria, with dcSSc involvement according to LeRoy and Medsger [8] were eligible if the following other criteria were met: (i) the skin involvement had to be significant with a modified Rodnan skin score [9] (mRSS) ≥15; and (ii) disease duration had to be <5 years since the first non-Raynaud phenomenon (RP) symptom or if longer, it was necessary to have a high mRSS and/or progression of skin involvement (mRSS increase of 3 or more points, new tendon friction rubs, and/or elevated inflammatory markers thought to be from worsening SSc). A complete list of the inclusion and exclusion criteria can be found in the Supplementary Appendix, available at *Rheumatology* online. All participants provided written informed consent. The study was approved by Health Canada (control number 214752).

### Procedures

Patients received intravenous brentuximab vedotin at a dose of 0.6 mg/kg every 3 weeks for a total of 45 weeks. This is the lowest effective dose studied in the phase 1 lymphoma trial that resulted in clinical responses, as no specific pharmacokinetic studies were available for SSc and it was the dose that Health Canada approved [10]. For this pilot study, no dose escalation was permitted. Additionally, participants were allowed to continue any preexisting standard of care treatment including cyclophosphamide, methotrexate, azathioprine, mycophenolate mofetil and/or mycophenolic acid. If treated with rituximab, the last dose needed to be at least 16 weeks prior to study entry.

During the trial, patients had outcome assessments (physician and patient-reported) at 0, 12, 24, 36 and 48 weeks. Laboratory tests were performed at each visit and a safety follow-up was done after the end of the study or after dropping out of the study. At each visit, screening for side effects including peripheral neuropathy was performed using a standardized form. Skin biopsies (distal extensor aspect of the forearm) were done at baseline, week 24 and weeks 45–48. Phone follow-up visits after finishing the infusions were done at weeks 52 and 56. The study exit visit was done at week 60. A data safety monitoring committee consisted of rheumatologists external to the study (Drs Lillian Barra, Sara Haig, Sherry Rohekar and Jason Lee, Western University, London, ON, Canada) and met every 6 months. All adverse events and serious adverse events were provided to the committee and discussed at the meetings. Meeting minutes were provided to J.E.P. Throughout the study, the institutional protocols implemented in our centre to prevent COVID-19 infection were followed.

### Outcomes

The primary end point was the change in the mean mRSS [9] from baseline to week 48. An improvement of ≥8 points in the mRSS was considered very clinically meaningful as the treatment was considered to have potential toxicity (serious infections, neuropathy), and a large patient commitment was required (visits every 3 weeks over 45 weeks for i.v. infusions and follow-up visits), the treatment was untested in SSc and patients with multiple drug failures were allowed to participate in the protocol (treatment-resistant and patients with severe disease were selected, such as a very high mRSS). The main secondary outcome was the change in mRSS from baseline to week. The other endpoints included: (i) change in mRSS from baseline to weeks 12 and 36; (ii) physician global assessment (MDGA) [11]; (iii) physician severity assessment (MDSA); (iv) physician damage assessment (MDDA); (v) scleroderma health assessment questionnaire-disability index (HAQ-DI) [12]; (vi) scleroderma health assessment questionnaire (SHAQ) [13]; (vii) functional assessment of chronic illness therapy-fatigue scale (FACIT); (viii) patient acceptable symptom state (PASS) [14]; and (ix) composite response index in diffuse cutaneous systemic sclerosis (CRISS) [15]. Bloodwork for efficacy and safety purposes was conducted before every study drug infusion including haemoglobin, leukocytes, neutrophils, platelets, erythrocyte sedimentation rate, c-reactive protein, alanine aminotransferase and glucose.

### Exploratory results

Additional mRSS were recorded from clinic visits following the study exit visit. Pulmonary function tests (PFTs) and doppler echocardiograms were performed as per standard of care (most recent before and after the study period). PFTs included forced expiration volume in one second (FEV1), total lung capacity (TLC) and diffusing capacity for carbon monoxide (DLCO). Serum and skin biopsy samples from participants were obtained unless the patient refused follow-up skin biopsies. Skin samples were obtained from the distal third of the forearms with two biopsies each time using a 3 mm punch biopsy using a sterile technique and sutures if needed at each of baseline, 24 and 48 weeks (with a window of ±2 weeks). Blood samples were frozen at -80°C and skin biopsies were preserved with formaldehyde. Inflammatory markers in the serum were explored including a commercial array. Immunohistochemical stains included haematoxylin, eosin, Lillie’s trichrome, CD4 and CD30. The laboratory and pathology specifications are available at the Supplementary Appendix, available at *Rheumatology* online. A semiquantitative assessment of the skin fibrosis (absent, mild, moderate, severe) was done by a dedicated pathologist (M.J.C.).

### Statistical analysis

The statistical analysis used R programming language and SPSS (Version 22, Chicago, IL, USA) for data processing and graphs. The sample size was estimated for 10 patients to detect a mRSS reduction of 8 points with a SD of 6 (*P* < 0.05; two-tailed, with 80% statistical power) and a 10% dropout. The sample size was calculated based on previous studies (SD 5.9 [16] and SD 7 [17]). The minimally clinical important difference (MCID) for mRSS after treatment based on a study with D-penicillamine was 3.2–5.3 [18]. The 8-point reduction threshold was chosen since the mean mRSS was predicted to be high with probable higher decreases (similar inclusion criteria to a study with imatinib with a mean mRSS of 32 [17]); to cover the possibility of a synergistic effect of concomitant immunosuppression; and to obtain a clinically meaningful response. Due to an early dropout of a patient who was hospitalized for acute pancreatitis, sample size was increased to 11 patients with approval from the ethics review board. The data analysis for the main primary and secondary endpoints (mRSS) was done using the intention-to-treat. The simple imputation method was used for missing values. The other variables were analysed per protocol. Mean, and standard deviation (SD) and frequency (%), paired sample Student’s *t*-tests, between groups differences used independent *t*-tests and Fisher’s exact tests were used for analyses.

## Results

### Baseline characteristics

Eleven patients were included in this study. The baseline characteristics are shown in Table 1. The ethnicity of the patients were White [9], Black [1] and Asian [1]. All patients had ANA ≥1:80 (4 speckled pattern, 4 nucleolar, 1 speckled and nucleolar, 1 centromere), two were positive for anti-topoisomerase antibodies, and one each was positive for anti-centromere antibodies, and anti-ribonucleic acid polymerase III antibodies. All patients had failed previous immunosuppression. The description of the previous immunosuppressive treatments, treatments received during the trial and changes in the primary outcome can be found in Table 2. All patients continued with their baseline immunosuppression (if any) throughout the study.

**Table 1.** Baseline characteristics of systemic sclerosis patients treated with brentuximab vedotin

	N = 11
Women	
*n* (%)	8 (72.7)
Previous immunosuppressive treatment	11 (100)
Interstitial lung disease	5 (45.5)
Age in years	
Mean (SD)	58.6 (13.5)
Disease duration in years	4.7 (3.4)
mRSS	30.2 (8.3)
FVC%	79.1 (26.6)
CRP mg/dL	3.7 (4.5)
PGA	6.2 (2.5)
MDGA	5 (2.1)
HAQ-DI	1.455 (0.799)
FACIT	25.4 (13.8)

CRP: c reactive protein; FACIT: functional assessment of chronic illness therapy-fatigue; FVC: forced vital capacity %; HAQ-DI: health assessment questionnaire-disability index; MDGA: medical doctor global assessment; mRSS: modified Rodnan skin score; N: number of patients; PGA: patient global assessment; SD: standard deviation.

**Table 2.** Individualized description and evolution of patients treated with brentuximab vedotin

Pt	Age (yrs)	Gender	Disease duration (yrs)	Past IS	Current IS	mRSS week 0	mRSS week 48	ΔmRSS week 48
1^a^	67	F	4.5	CYC, MTX, AZA, MMF	MMF	26	N/A	N/A
2^b^	59	F	5.7	MTX, AZA, ABA	None	36	19	−17
3	65	F	5	MTX	MTX	32	15	−17
4^b^	64	F	7.1	MTX, MMF	MTX	25	13	−12
5	45	M	3.5	GC, CYC, MTX, MMF	MMF	39	24	−15
6	68	M	1.5	GC, CYC, MTX	None	33	22	−11
7^a^^,^^b^	61	F	8	GC, CYC, MTX, MMF, AZA	AZA, NIN	18	N/A	N/A
8^b^	35	F	11.9	MTX, AZA, MMF	None	20	9	−11
9	38	F	3.2	MTX, MMF, RTX	MMF	23	22	−1
10	65	F	0.7	MTX	MTX	36	33	−3
11	77	M	0.6	MTX, MMF	MMF	44	29	−15

a Patient dropped out after hospitalization;

b inclusion due to persistent scleroderma activity with disease duration ≥5 years.

ABA: abatacept; AZA: azathioprine; CYC: cyclophosphamide; F: female; GC: glucocorticoids; IS: immunosuppression; M: male; MMF: mycophenolate mofetil; mRSS: modified Rodnan skin score; MTX: methotrexate; N/A: not applicable; NIN: nintedanib; Pt: patient; RTX: rituximab; yrs: years.

### Clinical endpoints

**Figure 1.**
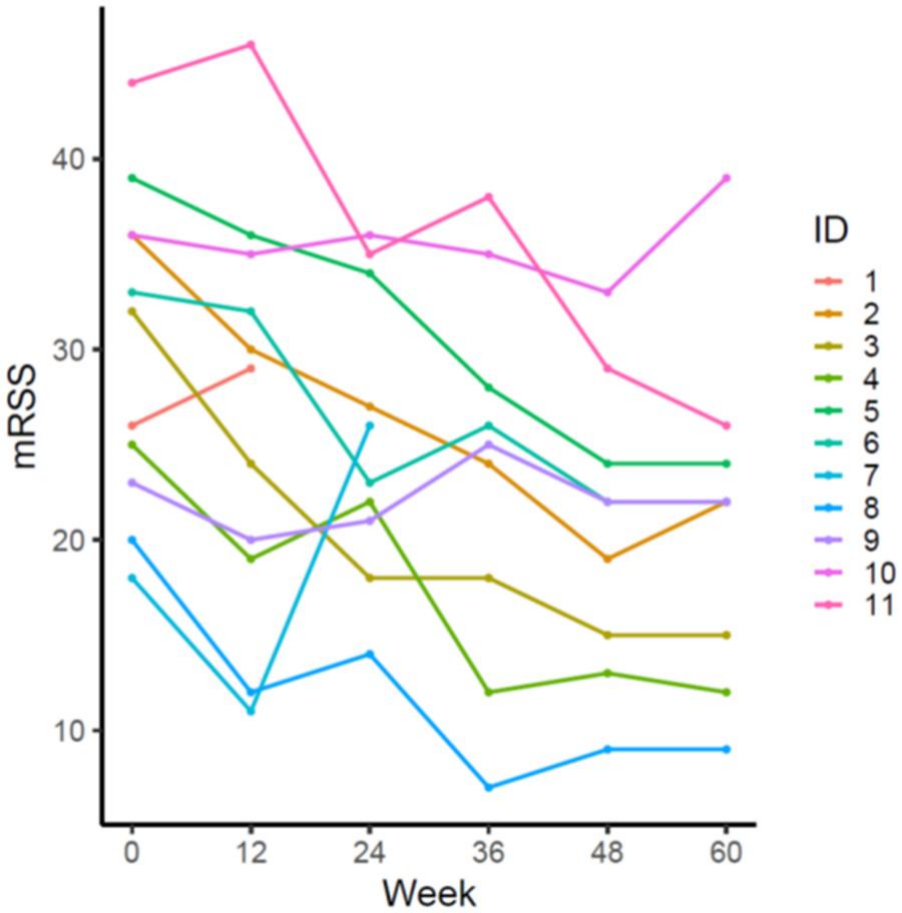
Evolution of the mean mRSS over time in patients treated with brentuximab vedotin. mRSS: modified Rodnan skin score

Nine of 11 patients completed the trial. The intention-to-treat analysis (11 patients) showed that the study met its primary end point with a mean mRSS reduction 8.3 points (from 30 to 22) at 48 weeks (Table 3). Additionally, the mRSS mean difference significantly decreased for all the interval periods (weeks 0–12, 0–24, 0–36, 0–48). The main secondary end point, which was a mRSS decrease of ≥8 points at week 24, was not met (mRSS reduction 4.3). ΔmRSS between weeks 0 and 48 was -11.3 (5.8). The evolution of mRSS values among patients is represented in Fig. 1. The week 60 data were collected at a mean of 62 weeks. The mRSS, off brentuximab vedotin seemed stable (21.9 at 62 weeks *vs* 20.7 at 48 weeks). The laboratory parameters did not change significantly during the study (Supplementary Table S1, available at *Rheumatology* online).

**Table 3.** Modified Rodnan skin score evolution for patients treated with brentuximab vedotin

mRSS Mean (SD)	N	Time 1	Time 2	Mean difference
Cases week 0–12	11	30.2 (8.3)	26.7 (10.7)	3.5 (95% CI 0.9, 6.1), *P* = 0.015
Cases week 0–24	10	30.2 (8.3)	25.9 (7.2)	4.3 (95% CI 0, 8.5), *P* = 0.05
Cases week 0–36	9	30.2 (8.3)	24.4 (9.1)	5.8 (95% CI 0.7, 11) *P* = 0.031
Cases week 0–48	9	30.2 (8.3)	21.9 (7.4)	8.3 (95% CI 2.5, 14), *P* = 0.01
Cases week 0–60	9	30.2 (8.3)	20.7 (9.2)	9.5 (95% CI 4.3, 15), *P* = 0.002
Cases week 48–60	9	21.9 (7.4)	20.7 (9.2)	1.2 (95% CI −2.9, 5.2), *P* = 0.5

CI: confidence interval; mRSS: modified Rodnan skin score; SD: standard deviation.

### Exploratory analyses

PFTs for the nine patients who completed the study were performed a mean of 28.7 (SD 21) weeks before study onset and at 55.5 (SD 14) weeks after the study start date. The FVC% predicted numerically trended to improve (Table 4) and ΔFVC% (FVC% week 0-FVC% week 48) was 7.8 (12.5).

**Table 4.** Pulmonary function tests in patients treated with brentuximab vedotin

N = 9 Mean (SD)	Before week 0	After week 48	Mean difference
FVC L	2.7 (1)	2.8 (1.1)	−0.1 (95% CI -0.2, 0.01); *P* = 0.071
FVC%	80.8 (23.8)	88.6 (26.2)	−7.8 (95% CI -17, 1.9); *P* = 0.1
FEV1 L	2.2 (0.8)	2.2 (0.9)	−0.07 (95% CI -0.2, 0.1); *P* = 0.4
FEV1%	81.1 (22.6)	88.6 (26.5)	−7.4 (95% CI -17, 2.2); *P* = 0.11
TLC L	4.4 (1.5)	4.7 (1.9)	−0.3 (95% CI -1, 0.4); *P* = 0.3
TLC%	85.4 (20.7)	87.9 (28.1)	−2.4 (95% CI -10, 5.4); *P* = 0.5

95% CI: 95% confidence interval; FEV1: forced ejection volume in 1 s; FVC: forced vital capacity; ΔFVC%: difference in forced vital capacity %; N: number; SD: standard deviation; TLC: total lung capacity.

Patient-reported outcomes (PRO) showed a significant improvement in the PGA and HAQ DI scores. Other outcomes are shown in Supplementary Table S2, available at *Rheumatology* online. The CRISS score at 48 weeks was 0.9 (0.4) (*N* = 7), with 86% of the patients reaching a meaningful CRISS score ≥0.6.

### Pathology and biomarkers

The proinflammatory marker study was compromised by the transient storage of the samples at -20°C instead of -80°C. As a result, soluble CD30, soluble interleukin 4 receptor, soluble receptor for advanced glycation end products, and soluble vascular endothelial growth factor receptors 1 and 3 were undetectable. None of the other measured markers were elevated at week 0 and they did not change at 48 weeks. Unfortunately, skin biopsies were compromised due to long-term storage with formalin. The CD30 staining was not interpretable (Supplementary Table S3 and Supplementary Fig. S1, available at *Rheumatology* online).

### Safety

Adverse events are reported in Table 5. No patients died during follow-up. Transaminase elevation (two patients), leukopenia (one patient) and eye pruritus (one patient) were the only ones considered to be related to the study drug administration. Only one patient (#1) developed SAEs. She had acute pancreatitis with gallstones 4 months after her treatment initiation and developed septic shock and pneumonia. The SAEs were considered unrelated to the study, but the patient decided to withdraw from the study. Another patient had moderate to severe preexisting ILD secondary to SSc. She developed right heart failure. She was diagnosed with pulmonary arterial hypertension, thought to be from her ILD and hypoxia. She was referred for PH management and a lung transplant. She withdrew from the study. Her mRSS in a follow-up visit equivalent to week 34 (7 weeks after the last infusion and on diuretics) was improved.

**Table 5.** Adverse events in patients with systemic sclerosis treated with brentuximab from week 0–60

Event	N (%)
Patients with any adverse event	8 (72.7)
Common adverse events	
Diarrhea	3 (27)
Oral ulcers	3 (27)
Pruritus	3 (27)
COVID-19 infection	2 (18)
Digital ulcers	2 (18)
Leukopenia	2 (18)
Elevated transaminases	2 (18)
Upper respiratory tract infection	2 (18)
Weight loss	2 (18)
Patients with severe adverse events	1 (9.1)
Severe adverse events	
Pancreatitis	1 (9.1)
*H. parainfluenzae* pneumonia	1 (9.1)

Adverse events with an incidence <10% were: rash, frozen shoulder, fever, epigastric pain, herpes zoster, hypotension, pitting oedema, wound infection, cellulitis, congestive heart failure, pulmonary hypertension, acute kidney injury, urinary tract infection, fatigue, generalized pain, eye pruritus, hypermenorrhea, left lower quadrant pain, pneumonia, hand tingling, anaemia, Raynaud’s phenomenon, dry skin, hemorrhoids. From this list, one patient had preexisting chronic mild leukopenia and one patient had preexisting hand tingling.

COVID-19: SARS-CoV-2 infection; N: number of patients.

## Discussion

In this open-label, single-arm, proof-of-concept study we have demonstrated that brentuximab vedotin significantly reduced the mean mRSS after 45 weeks of treatment. Patients also had an increase in the FVC. Brentuximab vedotin was beneficial for dcSSc skin involvement treatment. Furthermore, it may have benefit with respect to lung function and global assessments.

Considerations like study design, time since SSc diagnosis, duration of the study and the intrinsic limitations of mRSS are all potentially contributing to negative or slightly improved results in RCTs [4]. Compared with a 48-week phase III placebo controlled trial with tocilizumab, we recruited patients with a higher mRSS and a longer disease duration (mRSS 20.3 and <2 years *vs* 32 and 4.7, respectively) [16], which may or may not have skewed the results towards regression to the mean or less change in mRSS as the patients were already severe and past the traditional window of improving spontaneously and had failed many treatments. Our study allowed the concomitant use of other immunosuppressants whereas some other studies have used monotherapy with adding MMF only if there was significant worsening. mRSS at week 48 decreased by 6.1 points in the faSScinate trial, *vs* a nearly significant change compared with the placebo group (4.4). In the brentuximab trial, mRSS reduced by a mean of 8.3 points (*P* < 0.001) at 48 weeks.

Our study was not randomized, blinded or controlled. The CRISS outcome had a positive signal, as did FVC. The mRSS changes in this study seemed similar to the ones obtained at a meta-analysis pooling the results for stem cell transplant (−10.6) [18]. AHSCT is the most effective treatment for SSc skin involvement to date but indicated only in selected cases. Additionally, many patients do not have access to this treatment. AHSCT also has morbidity and mortality associated with the procedure and is not a cure.

Unfortunately, problems arising with sample storage prevented us from demonstrating the effects of brentuximab from a biomarker/pathology perspective. Authors acknowledge that larger studies should be conducted to clarify which subsets of patients with SSc have an upregulation of CD30 for a better understanding of the mechanism of action and to select those who could get a greater benefit from directed therapies. Aside from the potential benefits of treatment using the CD30 pathway, combining new therapeutic targets with other immunosuppressants that are used as the standard of care seems a reasonable way to obtain additional mRSS reductions. We did not compare those on monotherapy (*N* = 3) *vs* combination therapy in our study due to such a small sample size.

The safety profile with brentuximab vedotin on dcSSc patients did not show unexpected AE or SAE related to the study drug. It was generally well tolerated, and no patients died. The main SAE was an episode of pancreatitis, which has been described with this therapy in the absence of predisposing pancreaticobiliary problems and early in treatment [19]. In our case, the patient had received brentuximab for 4 months but she had gallstones as the likely cause. The same patient developed pneumonia during the admission, which was unrelated to the treatment as well. Brentuximab vedotin is known to cause peripheral neuropathy in patients where it is used at higher doses, generally in combination with chemotherapy agents [20]. This peripheral neuropathy generally appears between weeks 12 and 28 of treatment, it is dose-dependent, and it improves in 80% of the cases. One of our patients developed peripheral neuropathy in his feet >1.5 years after the final study visit. We don’t know if higher doses of brentuximab would yield more benefit and/or increased AEs. A separate dose-escalation phase I/II clinical trial is going on in the USA (NCT03222492, clinicaltrials.gov).

Different immunosuppressive drugs have shown improvements in FVC in clinical trials (cyclophosphamide [21] and mofetil mycophenolate [22]). Most recently, tocilizumab and nintedanib received FDA approval for prevention (tocilizumab) and reduction of worsening of pulmonary fibrosis (nintedanib). Brentuximab vedotin improved FVC%. Further studies are needed to determine whether brentuximab vedotin can favorably alter lung function.

Changes in PROs due to interventions have been extensively studied but results are inconsistent. Improvements have been reported on HAQ-DI (tocilizumab [16], AHSCT [23]), FACIT (tocilizumab [16]), MDGA (methotrexate [24], lenabasum [25]) and PGA (lenabasum [25]). Improvement of mRSS paralleled improvement in the PGA, HAQ-DI and MDGA.

The changes in mRSS might be magnified due to the higher baseline mRSS. However, these were patients with severe dcSSC as reflected by several patients having multiple drug treatment failures before study entry and high skin scores may be associated with worse outcomes. In the future, obtaining repeated biopsies from areas that are more likely to improve on treatment (proximal to elbows or knees, or abdomen) might have a higher yield in detecting fibrotic changes on skin biopsies.

## Conclusions

In summary, the results of this study showed that brentuximab vedotin improved mRSS in patients with dcSSc and met the primary end point of a mean change in mRSS of ≥8 points. Brentuximab vedotin seemed to improve skin scores and FVC. These findings provide a rationale for a large placebo-controlled trial of brentuximab vedotin added to standard of care in patients with severe active dcSSc.

## Supplementary material

Supplementary material is available at *Rheumatology* online.

## Supplementary Material

keae235_Supplementary_Data

## Data Availability

Supporting information is available in the Supplementary Appendix, available at *Rheumatology* online and further data is available at any time from the corresponding author on request, including de-identified patient data.
