# Supplementary material for: Brentuximab vedotin for skin involvement in refractory diffuse cutaneous systemic sclerosis, an open-label trial
Source: Rheumatology (Oxford). 2024 Apr 23;64(3):1476–81. doi: 10.1093/rheumatology/keae235 (PMC11879290; doi:10.1093/rheumatology/keae235)
Supplement: keae235_Supplementary_Data [file keae235_supplementary_data.zip › keae235_Supplementary_Data/rhe-24-0405-File008.pdf]

Re: ADCETRIS (Brentuximab vedotin) IIS submission

A pilot study of Adcetris treatment in active diffuse cutaneous systemic sclerosis (diffuse scleroderma)

Sponsor: This is an investigator-initiated study and Dr. Janet Pope is the sponsor. Seattle Genetics will be supplying drug and study funding.

Sites: St. Joseph's Health Care, London, ON (Dr. Pope) will be the only site.

Number of Participants: 10 patients

Submitted by Dr. Janet Pope, Professor of Medicine, UWO, London, ON
janet.pope@sjhc.london.on.ca

Running title: Pope – Brentuximab vedotin in early dcSSc

1 SYNOPSIS

Systemic sclerosis (SSc, Scleroderma) is a multisystem autoimmune disease characterized by widespread vascular injury and progressive fibrosis of the skin and internal organs. Internal organ involvement results in increased mortality of SSc patients. There is no effective treatment for the majority of patients with early active diffuse scleroderma (diffuse cutaneous systemic sclerosis; dcSSc). These patients early in their disease may be able to reverse their inflammation and reduce the probability of irreversible fibrosis via significant immune modulation. This is a pilot study that will treat 10 patients with early or active dcSSc who meet inclusion criteria to determine if the benefit of Brentuximab *vedotin* and safety are favorable in order to consider a randomized controlled trial. This is a Phase II study that is uncontrolled and patients will remain on their background immune suppressive treatment unless if contraindicated for safety or drug interactions. The trial is powered to show a mean change in mRSS of 8 over one year in an uncontrolled, unblinded study. The Health Assessment Questionnaire Disability Index (HAQ), patient and physician global scores, inflammatory markers, and combined response index in SSc (CRISS) will all be exploratory outcomes. Other outcomes such as changes in CD30-stained cells on skin biopsies with IHC from baseline to end of the trial will be explored if the study is positive.

2 BACKGROUND

2.1 Purpose

The purpose of this study is to assess feasibility, safety and preliminary efficacy of Brentuximab *vedotin* (Adcetris), a CD30-directed antibody-drug conjugate, in the treatment of active diffuse cutaneous systemic sclerosis (dcSSc).

2.2 Background and Rationale

Systemic sclerosis (SSc, Scleroderma) is a multisystem autoimmune disease characterized by widespread vascular injury and progressive fibrosis of the skin and internal organs. Internal organ involvement results in increased mortality of SSc patients, with cardiopulmonary complications being the most frequent causes of death [1]. The management of patients is challenging as there is no universal disease-modifying drug in SSc, and only few therapies have shown modest benefits in regard to some specific organ pathologies [2].

Immunosuppression is considered a cornerstone of therapy in rapidly progressive, active diffuse cutaneous SSc (dcSSc) [3]. Some of immunosuppressive agents (i.e. cyclophosphamide, methotrexate, rituximab) developed for anti-cancer therapy are being used in the treatment of SSc patients to improve the overall disease (skin score, interstitial lung disease, etc). Numerous connections between autoimmune rheumatic diseases and cancer have become increasingly evident over the past several decades. Compared with the general population, there is a two-fold increase in the risk of all invasive cancers in SSc patients, including hematologic neoplasms (SIR 2.6), such as non-Hodgkin's lymphoma (SIR 2.6) [4]. Some mutations in autoantigens initiate an autoimmune response against highly specific targets or in others hematologic malignancies [5]. Since the immune response to the autoantigen might be initiated and driven by the cancer and cross-reacts with antigens in normal tissue, effective therapy to remove the cancer could rid the host of the apical immune stimulus, and allow the peripheral immune-mediated damage to wane once resolution and tissue healing occur. The striking examples of autoimmune diseases disappearing after effective anticancer therapy [6-8] are consistent with this model. Additionally, autologous stem cell transplantation reduces skin fibrosis and organ involvement and after initial increased mortality (due to infection), there is a 3-year reduction in mortality compared to use of cyclophosphamide. This procedure is expensive and not well tolerated in SSc but implies that resetting the immune system and /or significant immune suppression can alter the natural history in severe early dcSSc. We hypothesize that novel cytotoxic agents for the treatment of hematologic malignancies such as Adcetris (brentuximab vedotin) may be beneficial in dcSSc.

Biologic rationale for use of Brentuximab vedotin in active dcSSc

SSc is an autoimmune disease characterized by inflammation and extracellular matrix deposition that ultimately leads to loss of organ function. T cells appear to play a prominent role in its pathogenesis [9,10]. The evidence for this comes from their being at the site of fibrosis, their activated phenotype and alteration in their number and frequency in peripheral blood [9,10,11]. SSc immune response is Th2-driven. Th2 cytokines are secreted in large quantities in SSc and they are promoting fibrosis and vascular activation/damage [11]. Activated Th2 cells also consistently and persistently express CD30 [12,13,14], a member of the tumor necrosis factor-receptor superfamily, and release measurable amounts of soluble CD30 (sCD30), both in vitro and in vivo [12,14,15]. CD30 expression appears to be restricted to fully differentiated Th2 cells and to other activated T cells that behave as Th2 precursors, whereas

committed Th1 effectors do not express CD30 [16,17]. The observations of high numbers of CD30+ T cells in the skin, and of high levels of sCD30 in the serum from patients with SSc [18,19], support the predominant activation of Th2 cells in SSc. Of note, high numbers of CD30+ T cells were found in the skin of the patient with graft versus host disease, which is also considered as a Th2-dominated disorder, and shares many similar clinical features with SSc [15,20]. By contrast, no CD30+ T cells were found in disorders with activated T cells showing a Th1 profile of cytokine production predominate (Crohn's disease) [15]. **The demonstration of a role for Th2-dominated response in the pathophysiology of SSc may have potential therapeutic applications.** Animal studies of Brentuximab in tight skin mice and other models of SSc-like fibrosis were not found in our literature search (no articles found in literature search).

The notion that T cells are involved in SSc is exemplified by the fact that treatment of a SSc patient with the humanized mAb alemtuzumab, targeting CD52, leads to a rapid and substantial clinical improvement [21]. Treatment with basiliximab, an anti-CD25 (surface marker of activated T-cells) antibody in SSc patients also resulted in improvement of skin thickening and pulmonary function, suggesting that T-cell targeting is a treatment option [22]. **Therefore, the strategies aimed at inhibiting the development and/or function of Th2 cells, such as anti-CD30 therapy, may be effective.**

Adcetris (brentuximab vedotin) is a CD30-directed antibody-drug conjugate consisting of three components: 1) the chimeric IgG1 antibody cAC10, specific for human CD30, 2) the microtubule-disrupting agent monomethyl auristatin E (MMAE), and 3) a protease-cleavable linker that covalently attaches MMAE to cAC10. Brentuximab vedotin delivers a cytotoxic agent that results in apoptotic cell death selectively in CD30-expressing cells, including activated T-lymphocytes, which is the primary mechanism of its action [20]. Targeted cytotoxic action of Brentuximab vedotin (anticancer activity and immunosuppressive properties) potentially represents an advantage over traditional therapy because it is designed to deliver the cytotoxic agent to specific cells (i.e., CD30-expressing tumor cells or pathogenic leukocytes), thereby resulting in an improved safety profile.

In summary, we hypothesize that activation of CD30-expressing T-cells in peripheral blood and tissue is related to disease activity in SSc, and targeted cytotoxic effect of Brentuximab vedotin on these cells (via disruption of the microtubule network within the cell by MMAE, cell cycle arrest and apoptotic death of the CD30-expressing cells) would result in improvement of clinical and pathological outcomes, such as reducing T-lymphocyte activation (sIL-2R serum level) and morphological signs of fibrosis (myofybroblast score in skin biopsies). However, the primary outcome will be a change in modified Rodnan skin score and if there is improvement in skin score, other parameters will be studied in detail including secondary outcome measures and lab measures (from serum and skin biopsies).

Use of background treatment

Many studies of unproven therapy in active dcSSc patients are added on to standard of care unless if there is a safety concern. For instance, a recent single site trial added belimumab or

placebo to mycophenylate mofetil (MMF). In many other rheumatic diseases such as systemic lupus erythematosus (another connective tissue disease), treatment is added to immune suppression such as background MMF, azathioprine or methotrexate [23] and even with rheumatoid arthritis and psoriatic arthritis, treatment is often studied in methotrexate inadequate responders. The treatment of SSc likely needs a combination of treatment (similar to lymphoma and other connective tissue diseases) as it is unlikely that one drug will give marked improvement in mRSS and other outcomes at least in the drugs studied to date.

3 MATERIALS AND METHODS

Subjects with active dcSSc will be enrolled after consenting and will receive courses of Brentuximab *vedotin* as an add on to standard of care which could include cyclophosphamide, methotrexate, azathioprine, mycophenylate mofetil (MMF, cellcept) and mycophenolic acid (myfortic). In some cases there will be no background immune suppressive such as patients with contraindications to specific medications or who have not responded to previous treatment and have ongoing active disease. Brentuximab vedotin will not be used in conjunction with current or recent rituximab.

4 CHOICE OF OUTCOME MEASURES

The primary outcome will be the change in mRSS.

The modified Rodnan Skin Score (mRSS) is a validated outcome measurement and is the usual primary outcome measurement for all trials of disease modification in scleroderma. It is positively correlated with internal organ involvement and has been shown to improve or worsen in many patients. The mRSS has good reproducibility and low inter and intra – rater variability [13-16]. The primary outcome assessor will always be the same person for each patient which will limit the variability.

Most contemporary trials in scleroderma use multiple outcomes: mRSS, MD and patient global assessments, quality of life, function (SHAQ and HAQ-DI) pulmonary function tests (FVC, DLCO), CRP, ESR and the CRISS (composite response index in SSc). Skin and serum are another way to study biomarkers for proof of concept [16,17].

The primary outcome for this study is the change in mRSS from the end of the treatment compared to the beginning to determine if there is any potential for Brentuximab vedotin to be further studied. This is an expensive and potentially toxic treatment so if there is not a large change in mRSS, the risk vs. potential benefit in further studies is likely not worth further pursuing of this treatment in dcSSc. In addition, this is an unblinded, uncontrolled study so there could be an additional 'placebo' effect of improvement and the drug under study will be added to standard of care, so there may be an additive effect of treatment where the component of improvement from standard of care will not be fully ascertained. The ethics of enrolling highly active dcSSc patients and not offering standard of care at this stage is

questionable and thus the study was designed to add Brentuximab vedotin to standard of care. This has been done in other studies such as imatinib [24] and belimumab (Clinicaltrial.gov identifier: NCT01670565) [25].

Justification for inclusion / exclusion criteria and outcome measurements
Diffuse cutaneous systemic sclerosis (dcSSc) has a higher morbidity and mortality than the limited subset with a far higher chance of developing serious internal organ involvement [26]. Most studies using immune modification in SSc study either organ involvement such as severe interstitial lung disease or skin involvement. Skin score correlated with morbidity – a worsening skin score as measured by the mRSS increases the likelihood of new or worsening internal organ involvement and an improving mRSS is correlated with stabilization, improvement or less worsening of internal organs (see abstract in appendix) [27]. Patients included in this study will be similar to other active dcSSc clinical trials [24, 25] as these are the patients with active disease who are thought to worsen without effective treatment. Inclusion criteria will reflect those seen in other studies of active dcSSc and exclusion criteria are primarily due to safety of Brentuximab Vedotin from the product monograph and other exclusion criteria for patients with marked other comorbidities or who are thought to have poor adherence.

Standard of care treatment in the subset with active dcSSc is somewhat anecdotal. Most are not candidates for autologous stem cell transplantation which increases survival but has initial morbidity and initial excess mortality associated with it and is not a cure [28,29]. Treatment is often with methotrexate, mycophenylate mofetil, azathioprine and/or cyclophosphamide (see abstract in appendix) [30]. The mean change over one year in skin score is often only a few points over a year. Even a multi-site trial with tocilizumab demonstrated only a minor improvement in mRSS at one year compared to placebo with a change with placebo of 2.77 mRSS improvement (and some patients were able to escape at 6 months and add background immune suppression) compared to 6.33 improvement in mRSS with tocilizumab [31]. One year of oral cyclophosphamide treatment improved the skin score by 6.1 at the end of two years vs. MMF with only a 2.9 improvement where patients were mostly early dcSSc with ILD but 1/3 had lcSSc (where the mRSS would not change much during the trial) [32].

Rationale for a calculated 8 point change in mRSS as the primary outcome
Virtually all trials in active dcSSc use the modified Rodnan Skin Score (mRSS) as the primary outcome due to its reliability, and ability to change with effective treatment and relationship to internal organ involvement. The patients chosen will have a slightly higher mean mRSS than the tocilizumab study as it is anticipated that the baseline mRSS will be approximately 32 (as this is similar to the imatinib study which had virtually the same inclusion criteria) and background therapy is allowed in this protocol and was not allowed for the first 6 months in the tocilizumab trial [31,24]. Experts have agreed that an improvement in mRSS of at least 5 points is clinically relevant [33] so we have chosen an improvement of 8 points on the mRSS in order to have adequate sample size (see sample size justification) and be relevant to scleroderma experts and know that this is the ‘best possible treatment effect’ as the treatment will be added to standard of care so there may be additive or synergistic improvement (as we assume that there should

not be interference of background treatment with Brentuximab vedotin due to the lymphoma protocols allowing for background immune suppression).

Justification of dosage
There is a trial with Brentuximab vedotin underway in Ann Arbor, Michigan with up-dosing patient groups. This is a safety study and no data will be analyzable for improvement in the mRSS. The purpose of our protocol is to perform repeat dosing at the lowest dose approved in lymphoma in order to determine if there is efficacy over 48 weeks with respect to the modified Rodnan skin score (mRSS). In order for the protocol to test only one thing, the treatment will remain stable with repeat dosing unless if there is a safety concern and then treatment will be reduced as per the product monograph. It is common in SSc (often due to the rarity and orphan status) to 'borrow' dosing from other diseases such as with rituximab (dose is borrowed from rheumatoid arthritis and belimumab (dosing is the same as SLE) [25,34]. There have not been formal pharmacokinetic studies within SSc with Brentuximab vedotin.

The protocol could be amended if good safety has been demonstrated with Brentuximab vedotin from the Ann Arbor study or if our current study shows good safety in the initial 10 patients and modest efficacy with respect to the mRSS. However, likely a properly designed RCT would be developed if the primary end point of this study is achieved and if there is good tolerability.

Safety in SSc patients with treatment may be different than in other diseases. For instance in the tocilizumab dcSSc study, the serious infection rate was far higher than in RA for both the placebo and the active treatment groups. Thus the lowest dose for Brentuximab vedotin was chosen (although it has been tested in patients with lymphoma who were often exposed to other strong immune suppressive drugs) (product monograph) [35].

Safety monitoring
The protocol has laboratory monitoring for safety from the Brentuximab vedotin product monograph. All dose adjustments will be via the product monograph and stopping rules in the product monograph will apply in this study.

The interaction between standard of care immune modulators and Brentuximab vedotin is unknown in SSc but it is important to perform this study in addition to standard of care as denying standard of care in this highly active population is not good clinical practice and we don't know if there is any benefit using Brentuximab vedotin. Also the efficacy on skin is at the earliest 6 to 12 months in other active dcSSc patients so with holding standard of care is inappropriate for ethical reasons.

Data Safety Monitoring Board (DSMB)
There will be the protection of human subjects using the ICH guidance for clinical trials. A Data Safety Monitoring Board (DSMB) will consist of two rheumatologists who are at arm's length from the trial (Dr. Lillian Barra and Dr. Jason Lee) and they will monitor the safety every 6

months and also when 5 patients have been treated for at least 6 months in order to determine if the study should be continued. We expect many AEs and more serious infections in SSc than in for example rheumatoid arthritis (where the serious infection rate is approximately 3 to 5 per 100 patient years [36] as it was higher with tocilizumab in dcSSc than in RA [31,37].

The study will be considered a go/no go where if there is not at least a mean change of 8 points improvement on the mRSS in this poor prognosis population of active dcSSc patients, then due to the potential risks of the study drug and potential other treatments that may be available and approved by Health Canada, Brentuximab Vedotin will not be further studied. The purpose of this proposal is to inform both safety and efficacy for a properly designed (double blinded placebo controlled, added to background standard of care) phase II study in the future.

5 STUDY DURATION

The duration of this study is up to 64 weeks, including a screening period of up to 4 weeks, study visits and infusions every 3 weeks for 48 weeks, and a follow-up visits at Weeks 52 and 56 (both are telephone calls) and at Week 60 (a final visit) after last dose of brentuximab *vedotin*.

This is an uncontrolled study comparing the post treatment mRSS within each participant from the pre treatment value.

6 SELECTION AND WITHDRAWAL OF PARTICIPANTS

Inclusion Criteria:

1. Patients with scleroderma, aged 18 years or older, and:

2. Subjects must meet the ACR/EULAR classification criteria for SSc (2013) [18].

3. Early dcSSc (within 5 years of first non-RP symptom) or active dcSSc as determined by worsening mRSS, presence of tendon friction rubs, and/or elevated inflammatory markers thought to be due to active dcSSc and not related to other issues such as infection.

4. Minimum modified Rodnan Skin Score of 15 (range, 0-51, with higher scores indicating more severe skin thickening) [19].

5. Able to give informed consent.

6. A negative TB skin test at screening, or treatment with INH for 6 months or other standardized LBTI (latent TB infection) treatment in the past.

Exclusion Criteria:

1. Poor pulmonary function (FVC<40% and/or DLCO<30%).

2. Pregnancy, breast feeding or child bearing potential without practicing reliable contraception (and partners for men in the study).

3. Clinically significant pulmonary hypertension requiring drug therapy.

4. Clinically significant cardiac disease.

5. Chronic or ongoing active infectious disease requiring systemic treatment.
6. Seropositivity for human immunodeficiency virus (HIV) at study entry.

7. Active tuberculosis (TB) infection.

8. Active viral infection with viral replication of hepatitis B or C virus at study entry.

9. Significant concurrent, uncontrolled medical condition including, but not limited to, renal, hepatic, pancreatic, haematological, gastrointestinal, endocrine, pulmonary, neurological, cerebral or psychiatric disease; and cancer.

10. Peripheral neuropathy at screening Grade 2 or higher.

11. Known or suspected hypersensitivity to components of the treatment

12. Patients known or suspected of not being able to comply with a study protocol (e.g. due to alcoholism, drug dependency or psychological disorder)

13. Any of the following laboratory abnormalities at screening:
- Absolute neutrophils count <2.0 x 10^9/L
- Hemoglobin <85 g/L
- Platelet count < 100 x 10^9/L
- AST/SGOT or ALT/SGPT >2.0 UNL

14. Participation in another clinical trial within six weeks before randomization in this study

15. Use of rituximab within the previous 4 months.

16. Immunization with a live/ attenuated vaccine less than 4 weeks prior to the baseline visit.

17. Previous use of brentuximab vedotin.

18. Current or history of progressive multifocal leukoencephalopathy (PML).

<u>Discontinuation of Study Participants from Treatment</u>

Participants must discontinue Brentuximab Vedotin for any of the following reasons (many items reflect the product monograph):
-Withdrawal of informed consent. The patients are free to withdraw consent or drop out at any time with no adverse effect on their future care.
- Any adverse event (serious or non-serious) or concurrent illness that, in the opinion of the investigator, indicates continued use of Brentuximab Vedotin to not be in the best interest of the participant.
-Pregnancy
-Use of prohibited medications or live vaccines

All patients who discontinue treatment in this study should be followed for the duration of the study, complying with all study related procedures, except administration of study drug, as per the Schedule of Assessments. (Table D) If a participant withdraws from the study prior to the study completion, the reason for withdrawal must be well documented.

7 STUDY OBJECTIVES AND OUTCOMES

Primary Objective
Change in skin thickening over time measured by modified Rodnan Skin Score at 12 months (12 months mRSS minus baseline mRSS for each participant). A paired t test P-value will be done. The mRSS at 6 months will be a secondary outcome.

Outcome measures:
<u>Primary Outcome Measure</u>: Change in skin thickening over time measured by modified Rodnan Skin Score at 12 months.

<u>Other Outcome Measures:</u>
A key secondary endpoint will be mRSS at 6 months.

<u>Secondary and Exploratory Outcome Measures</u>:

- Change in skin thickening over time measured by mRSS at 3 and 9 months
- Changes in physician-assessed health status at 3, 6, 9 and 12 months. Physician global assessment of disease activity on VAS: “How active would you rate the patient scleroderma for the past week?” ranked from 0 to 10 with 10 being the most active disease. Additionally, the physician global assessment of disease severity and damage will be measured by using VASs ranked from 0 to 10 with 10 being the most severe disease or the most pronounced damage, respectively.

- Changes in patient reported outcomes (PROs) at 3, 6, 9 and 12 months will be assessed including patient global assessment of disease activity (0 to 10), Health Transition score ("Compared to 6 months ago, how do you rate your health overall?" on a 1-5 scale with 1 being much worse, 2 – worse, 3 same, 4 – better, 5 – much better) and the Scleroderma Health Assessment Questionnaire (SHAQ) is one of the most widely used and best characterized outcome measure in SSc. It combines the disability index of the (Health Assessment Questionnaire) HAQ, which is widely validated for other rheumatic diseases with a series of visual analogue scales (VAS) specifically related to SSc. The SHAQ has been shown to be both cross-sectionally and longitudinally reliable.
- Change in pulmonary function (time frame: 6 and 12 months)

  % Improving/worsening FVC and DLCO. Analyzed as an ordinal outcome.
- CRISS measurements at baseline and to define disease progression at 6 months [9].
- Change in serum concentrations of acute phase reactants at 3, 6, 9 and 12 months (ESR, hsCRP)
- Regimen-related toxicities (up to 12 weeks post-treatment)

  Defined as adverse events (AEs) >= Grade 3 and assessed by the investigator as 1 of the following: related or unrelated to treatment.
- Infectious complications (time frame: assessed up to 1 month post-treatment)
- Change in peripheral levels of CD30 marker and sIL-2R (3, 6, 9 and 12 months) and CD30-positive cell count in skin biopsies of involved forearm skin at at 6 and 12 months (12)
- Changes in serum aminoterminal propeptide of type III collagen levels . (3, 6, 9 and 12 months), and myofibroblast score in skin biopsies (6 and 12 months) if the study looks favorable with respect to potential benefit and safety

8 INVESTIGATIONAL MEDICINAL PRODUCT ADMINISTRATION of Brentuximab Vedotin

In this study, brentuximab vedotin is the investigational medicinal product. Brentuximab *vedotin* will be given using the following guidelines. We will start at low dose of brentuximab vedotin (0.6 mg/kg) for safety reasons intravenously every 3 weeks for a maximum of 16 cycles (48 weeks). This is the lowest dose in the product monograph and will not be escalated in this protocol for safety reasons (and will need an amendment to escalate dosing dependent on the data from the Phase 1b study in SSc performed at Ann Arbor).

All randomized participants in this study will receive open-label treatment at 0.6 mg/kg intravenously every 3weeks, as tolerated. See Table A and Table B for dose decreasing guidelines.

Dosing will occur based on results of safety blood work done prior to each dose of brentuximab *vedotin*. (see Table A and Table B) Median duration of treatment in hematologic protocols

seems to be 24 to 27 weeks (range, 3 to 75 weeks) with a maximum of 16 doses, therefore, in this protocol we will administer up to 16 infusions every 3 weeks (48 weeks) of brentuximab *vedotin* added to standard of care.

Dosing is based on patient weight. Actual weight will be used except for patients weighing greater than 100 kg; the dose for patients with weight greater than 100 kg will be calculated based on 100 kg. Rounding is permissible within 5% of the nominal dose.

Table A Decreases in Neutrophil Count

Neutrophil (x 10^9/L)	**Action**
≥ 0.5	May continue 0.6 mg/kg
< 0.5	Hold dose until count ≥ 0.5 and may decrease dose as appropriate

Table B Decreases in Platelet Count

**Platelet Count** (x 10^9/L)	**Action**
≥ 50	May continue to dose at 0.6 mg/kg
<50	Hold dose until platelet count ≥ 50 and may decrease dose as appropriate

According to the grading criteria, neuropathy characterized by (for sensory) loss of deep tendon reflexes or paresthesia or (for motor) weakness on exam or testing that does not interfere with function is assigned Grade 1; neuropathy that interferes with function, but not activities of daily living is assigned Grade 2; neuropathy that interferes with activities of daily living is assigned Grade 3, and neuropathy that is disabling is assigned Grades 4, respectively. As per the product monograph, if there is Grade 4 neuropathy then the drug must be full discontinued.

Preparation of Brentuximab Vedotin

Vial Storage and Handling

Refrigeration should be set at 2–8°C for storage of vials and solutions containing brentuximab vedotin.

Chemical and physical stability of the reconstituted brentuximab vedotin drug product has been demonstrated for 24 hours at 2–8°C and 25°C. However, brentuximab vedotin does not contain preservatives; therefore, from a microbiological standpoint, opened and reconstituted vials should be used immediately. If not used immediately, the in-use storage should not be longer than 24 hours. It is recommended that brentuximab vedotin vials and solutions be protected from direct sunlight until the time of use.

Handling Precautions

Recommended safety measures for the handling and preparation of brentuximab vedotin for Injection include masks, protective clothing, gloves, and vertical laminar airflow safety cabinets.

Reconstitution

1. Reconstitute lyophilized brentuximab vedotin by adding 10.5 mL Sterile Water for Injection, USP, or equivalent to the 50 mg vial, directing the stream to the side of the vial. The concentration of reconstituted brentuximab vedotin is 5 mg/mL with a total volume of 11 mL.
2. Gently swirl the vial until contents are completely dissolved. The vial must not be shaken. Slight "bubbling" of the solution upon reconstitution may be observed.
Pharmacy Instructions Version 10; 16-Oct-2017
Brentuximab vedotin Seattle Genetics, Inc. – Confidential Page 5 of 6
3. Allow the reconstituted vial to settle for a minute to allow bubbles to dissipate. The reconstituted product should be a colorless, clear to slightly opalescent solution with no visible particulates.
4. Refrigeration should be set at 2–8°C for storage of the reconstituted vials. The reconstituted vials must be administered within 24 hours of vial reconstitution. Protect infusion bag from direct sunlight until time of use.

Dose Preparation

1. Transfer the required volume of reconstituted product to a 100 mL infusion bag. It is recommended that the infusion bag be labeled with the patient number or name, and the drug name and lot number (or kit number in the case of a blinded study). The infusion bag size should allow enough diluent to achieve a concentration of 0.6–1.8 mg/mL. The following bag types are compatible with brentuximab vedotin: polyvinylchloride (PVC), ethylene vinyl acetate (EVA), polyolefin, polypropylene, or polyethylene. Discard any remaining reconstituted product.
2. Dilute reconstituted product in either 0.9% Sodium Chloride Injection, Lactated Ringer's solution, or dextrose 5% in water (D5W). The diluents should be USP grade or equivalent. The final concentration of brentuximab vedotin in infusion bag should be in the range of 0.4–1.8 mg/mL. See example calculations in Table 1 below.

- Total dose = patient weight x dose level. Note that for patients weighing more than 100 kg, total dose will be calculated using 100 kg.
- Volume of reconstituted product required = total dose/5 mg per mL
- Final concentration = total dose/total volume of infusion

Table C – Note the dose will not exceed 0.6 mg/kg

**Examples of dose preparation calculations** Pt weight (kg)	Dose level (mg/kg)	Total dose (mg)	Volume of reconstituted product required (mL)	Volume of diluent (mL)	Total volume of infusion (mL)	Concentrationa (mg/mL)
45	1.2	54	10.8 (requires 2 vialsb)	100	110.8	0.49
70	1.8	126	25.2 (requires 3 vialsb)	150 or 250	175.2 or 275.2	0.72 or 0.46
120	1.8	180c	36 (requires 4 vialsb)	150 or 250	186 or 286	0.97 or 0.63

a Must be between 0.6 and 1.8 mg/mL
b Approximately 10 mL can be withdrawn from each vial.
C For patients weighing more than 100 kg, total dose will be calculated using 100 kg.

3. Gently invert the infusion bag. DO NOT SHAKE or expose the prepared dosing solution to excess vibration at any time when transferring or transporting the reconstituted product. Pneumatic tube systems are not recommended.
4. Prior to administration, inspect the prepared dosing solution (in infusion bag) for any particulate matter or discoloration.
5. Do not prepare a single dose of brentuximab vedotin using vials from different lots or kits. Use vials from the same lot or kit number for a given dose.
6. Refrigeration should be set at 2–8°C for storage of the prepared dosing solution. The solution must be used within 24 hours of vial reconstitution. Protect infusion bag from direct sunlight until time of use.

BRENTUXIMAB VEDOTIN MUST NOT BE ADMINISTERED AS AN IV PUSH OR BOLUS. Brentuximab vedotin should be administered over approximately 30 minutes and cannot be mixed with other medications.

Infusion Monitoring Guidelines

-Blood pressure, heart rate, respiratory rate and temperature will be monitored pre-infusion, post-infusion, then every 30 minutes x 2 post the first infusion and every 30 minutes x 1 post the second and third infusions.

-If an infusion-related reaction occurs, the infusion should be interrupted and appropriate medical management instituted. Monitor every 10 minutes until reaction resolved. Subsequent infusions should be discontinued or given with premedication (acetaminophen (500-1000 mg orally), antihistamine (diphenhydramine 25-50 mg orally or intravenously) and corticosteroid (methylprednisolone 50-100 mg intravenously), dependent on the severity of the reaction.

-If anaphylaxis occurs, immediately and permanently discontinue administration and administer appropriate medical therapy.

Drug Ordering and Accountability

Brentuximab vedotin will be supplied by Seattle Genetics, Inc. and administered to the patients under standard of care.

Drug Disposal

Any partially used vials or prepared dosing solutions may be discarded in accordance with institutional drug disposal procedures. Partially used vials may not be used to provide treatment for a different patient. See the Pharmacy Binder for instructions for disposing of unused vials.

9 CONCOMITANT MEDICATIONS AND TREATMENTS

Study participants will be allowed to continue taking their medications for underlying comorbidities. The treatment with brentuximab vedotin is in addition to standard of care medications for systemic sclerosis and may include cyclophosphamide, methotrexate, azathioprine, mycophenylate mofetil (MMF, cellcept) and mycophenolic acid (myfortic). Treatment for GERD, Raynaud's, digital ulcers, HTN, delayed GI emptying, small bowel overgrowth, Sjogren's symptoms, and arthritis (ex. using NSAIDs) are allowed.

Severe PAH and severe ILD is excluded as there could be expectations that this comorbidity may interfere with the treatment protocol.

Low dose steroids (10 mg/d daily prednisone maximum) are allowed but if a patient needs high dose, this will be a protocol violation and noted as a treatment failure.

Supportive care. Other drugs being used to prevent side-effects or/and anti-drug antibody formation as per standard of care: such as acetaminophen (Tylenol) (500mg, given as a single oral dose 30 minutes prior to infusion), diphenhydramine (Benadryl) (25-50mg, given as a single oral/intravenous dose 30 minutes prior to infusion).

Good clinical practice will be followed and other appropriate treatment will not be denied. Concomitant medications will be assumed to be held stable throughout the study where ever possible.

Prohibited Medications/Treatments

Immunosupressive drugs other than cyclophosphamide, methotrexate, MMF, and azathioprine must be discontinued and are not allowed throughout the study.

Patients will be asked to avoid grapefruit juice around their infusions as this could potentially increase the dose of brentuximab vedotin.

Concomitant use of brentuximab vedotin with strong CYP3A4 inhibitors and/or P-gp inhibitors will be closely monitored for adverse reactions due to increase in exposure.

10 STUDY ASSESSMENTS AND PROCEDURES

The following assessments and procedures will be performed for this study, as per Schedule of Assessments (Table D)

-Informed Consent: Study information will be provided to the patient, both orally and written, and informed consent will be obtained prior to any study procedures.

-Medical History: General medical and surgical history, concomitant medical diseases/ conditions and medication history will be obtained.

-Physical Examination: A standard physical examination will be performed by the physician. Abnormal assessments made at screening will be considered a part of the patient's medical history. Any new, abnormal findings observed after the screening visit will be considered an adverse event. Neurological examination of upper and lower limbs will be done prior to each infusion to rule out a peripheral neuropathy as a possible side effect of Brentuximab vedotin.

-Modified Rodnan Skin Score (mRSS): This is a measurement of skin thickness, with a scoring of a 0 (normal) to 3+ (severe induration) over 17 body areas, with a maximum score of 51.

Changes in:
  -mRSS from the end of treatment minus baseline is the primary outcome

-Vital Signs: This will include heart rate, blood pressure, respiratory rate, temperature, height (at screening only) and weight.

-CRISS (Composite Response Index Systemic Sclerosis): This is an evaluation of a patient's response to treatment and includes evaluations for
  -New scleroderma renal crisis
  -Decline in forced vital capacity (FVC) % predicted ≥15% with repeat PFTs confirming Interstitial Lung Disease (ILD)
  -New onset of left ventricular failure
  -New onset of pulmonary arterial hypertension (PAH) on right heart catheterization requiring treatment

  -Patient Global Assessment of disease activity
  -Physician Global Assessment of disease activity
  -HAQ-DI
  -Forced Vital Capacity (FVC)

-Pulmonary Functions Test: This will be done under standard of care

-ECG- under standard of care

-Echocardiogram: done as part of standard of care

-Chest Xray: done as part of standard of care

-Lab Testing: Full safety evaluation including CBC and differential, electrolytes, biochemistry (liver and renal function tests, glucose level), ESR and hsCRP will be done at Screening and at Months 3, 6, 9 and 12.

As part of standard of care (SoC) prior to each infusion, CBC with differential and blood chemistry (including ALT, total bilirubin, creatinine and glucose) will be performed to monitor

for toxicity looking Grade 3 or 4 neutropenia where the dose will be held or adjusted according to product monograph. (see Tables A and B)

- Adverse Events: Patients will be assessed for adverse events at each study visit.

-Concomitant Medication Review: All concomitant medications taken by the patient, prescription and over-the-counter medications and supplements, will be reviewed at each visit.

- Serum and skin biopsies: will be collected for investigating collagen turnover and inflammation. Blood samples will be taken at baseline, 3, 6, 9 and 12 months and analyzed by ELISA/multiplexing for changes in serum sIL-2R, sCD30 and aminoterminal propeptide of type III collagen levels. Two 3mm skin biopsies, to measure CD30 stained cells and myofibroblast count, will be taken from the forearm prior to the first cycle, at 6 and 12 months.

<u>Table D: SCHEDULE OF ASSESSMENTS (TREATMENT PERIOD)</u>

	Screening -28 to -1	Baseline Week 0	Week 3	Week 6	Week 9	Week 12	Week 15	Week 18	Week 21	Week 24	Week 27	Week 30	Week 33	Week 36	Week 39	Week 42	Week 45	Week 48
Informed Consent	X																	
Inclusion/Exclusion	X																	
Medical History	X																	
Demographics	X																	
Physical Exam	X	X				X				X				X				X
Vital Signs	X	X	X	X	X	X	X	X	X	X	X	X	X	X	X	X	X	X
Signs/Symptoms Assessment		X	X	X	X	X	X	X	X	X	X	X	X	X	X	X	X	X
MRSS/ DU Count	X	X				X				X				X				X
CRISS Assessment[b]		X								X								X
PFTs [a]	X									X								X
Echocardiogram[a]	X									X								X
ECG[a]	X																	
Chest Xray[a]	X																	
Skin Biopsies[c]		X								X								X
Research Bloods		X				X				X				X				X
ESR, hsCRP	X	X				X				X				X				X
CBC + diff	X	X	X	X	X	X	X	X	X	X	X	X	X	X	X	X	X	X
ALT, Total Bilirubin, Creatinine, Glucose	X	X	X	X	X	X	X	X	X	X	X	X	X	X	X	X	X	X
Hepatitis B & C, HIV	X																	
AST	X																	
Pregnancy test (as applicable)[d]	X	X	X	X	X	X	X	X	X	X	X	X	X	X	X	X	X	X

Patient PROs[e]		X				X				X				X				X
MD Global		X				X				X				X				X
Concomitant Medications	X	X	X	X	X	X	X	X	X	X	X	X	X	X	X	X	X	X
Adverse Events	X	X	X	X	X	X	X	X	X	X	X	X	X	X	X	X	X	X
Brentuximab infusion*		X	X	X	X	X	X	X	X	X	X	X	X	X	X	X	X	X

* If Brentuximab infusions discontinue, the rest of assessment visits will be still done every 12 weeks accordingly to the schedule week 12, 24, 36 and 48.
[a] Data will obtained from tests done within 3 months prior to screening visit
[b] CRISS includes: MRSS, Patient and MD Global Assessments, FVC, Assessment for Scleroderma Renal Crisis, Decline in FVC ≥15%, New onset Left Ventricular Failure requiring treatment and new PAH
[c] Two 3-mm skin biopsies will be collected.
[d] For women of child-bearing potential. Serum or urine.
[e] PROs include: Patient Global Assessment, SHAQ (HAQ DI and VAS scales), FACIT, EQ-5D, Patient Acceptable Symptom State (PASS)

Table E: SCHEDULE OF ASSESSMENTS – Follow-Up Period

	Week 52 (telephone call)	Week 56 (telephone call)	Week 60
Vital Signs			X
Signs/Symptoms Assessment	X	X	X
Concomitant Medications	X	X	X
Adverse Events	X	X	X
CBC + diff			X
ESR, CRP			X

11 ADVERSE EVENT REPORTING

Adverse Event

An adverse event (AE) is defined as any untoward medical occurrence experienced by a research participant administered an investigational product and which does not necessarily have a causal relationship with this product. An AE can therefore be any unfavorable and unintended sign (including an abnormal laboratory finding, for example), symptom, or disease temporally associated with the use of an investigational product, whether related or not to the investigational product (CAREB AE Guidelines, July 2010).

Assignment of Adverse Event Intensity and Relationship to Brentuximab Vedotin

This study will use the CTCAE (NCI Common Terminology Criteria for Adverse Events) versions 4.0 for adverse event reporting. Grade refers to the severity of the AE. The CTCAE displays Grades 1 through 5 with unique clinical descriptions of severity for each AE based on this general guideline:

Grade 1 Mild; asymptomatic or mild symptoms; clinical or diagnostic observations only; intervention not indicated.

Grade 2 Moderate; minimal, local or noninvasive intervention indicated; limiting age-appropriate instrumental ADL*.

Grade 3 Severe or medically significant but not immediately life-threatening; hospitalization or prolongation of hospitalization indicated; disabling; limiting self-care ADL**.

Grade 4 Life-threatening consequences; urgent intervention indicated.

Grade 5 Death.

*Instrumental ADL refer to preparing meals, shopping for groceries or clothes, using the telephone, managing money, etc.
**Self care ADL refer to bathing, dressing and undressing, feeding self, using the toilet, taking medications, and not bedridden.

The following categories and definitions, as determined by the physician, will be used in this study to report causal relationship:

Related: There is reasonable causal relationship between the administration of study drug and the adverse event.
Not Related: There is not a reasonable causal relationship between the administration of study drug and the adverse event.

Serious Adverse Event

Serious Adverse Event/Experience (SAE) or Reaction is defined as any untoward medical occurrence that:
• results in death
• is life-threatening
• requires inpatient hospitalization or prolongation of existing hospitalization
• results in persistent or significant disability/incapacity
• results in a congenital anomaly/birth defect
• based upon appropriate medical judgement, is an important medical event that may jeopardize the health of the research participant or may require medical intervention to prevent one of the outcomes listed above.

Note: The following hospitalizations will not be considered SAEs for this study:

- A visit to the emergency room or other hospital department lasting less than 24 hours that does not result in admission (unless considered an "important medical event" or a life-threatening event)
- Elective surgery planned before signing consent
- Admissions for a planned medical/surgical procedure

Serious adverse events will be reported from the time the participant signs written, informed consent until 30 days following last dose of study medication.

Collection and reporting of AEs and SAEs
All study participants will be assessed at each study visit for safety, and will be encouraged to notify Dr. Pope or the research staff of any symptoms, illnesses or changes to their health. Safety monitoring will include adverse events, clinically significant changes to vital signs and laboratory test abnormalities. Signs and symptoms of adverse reactions to the study drug will also be monitored. Participants will be instructed and encouraged to report any serious adverse event as soon as possible to the study site. AEs and SAEs will be collected and reported from the time of signing consent until the final follow-up visit has occurred.
SAEs that are determined by Dr. Pope or a Sub-investigator to be related to brentuximab vedotin will be reported, as required, to Health Canada.

Safety Reporting Requirements:

Reporting Timeframe: The Principal Investigator will report all Serious Adverse Event (SAE)s that occur in a study subject within the following timeframe:

Individual expedited SAE reports required by competent health authority (Health Canada)
At time of submission to competent authority:
Seattle Genetics Drug Safety by: Facsimile (425) 527-4308 or (866) 333-6627 (USA only toll free)
Email: drug.safety@seagen.com

Aggregate listing of all SAEs
Monthly:
IST@seagen.com or portal

Pregnancies from time of first drug until 6 months after last dose, including any that occur in male patient's partner.

Within 48 hours of awareness, email or fax Seattle Genetics pregnancy report form to Drug Safety. Abortion, whether accidental, therapeutic or spontaneous should be reported as an SAE

Reporting Forms for individual expedited submissions: The Principal Investigator will report such SAEs on the approved local regulatory form (i.e. FDA MedWatch form or CIOMS) to include an assessment of causality to the Product.

Reporting format for monthly SAEs: The ISTSponsor-Investigator will provide a study-specific cumulative SAE line listing of all SAEs including an assessment of causality to the Product.

Reporting Period: The reportable events that are subject to this provision are those that occur from the start of administration of the first dose of the Product through thirty (30) days after discontinuation of the Product. SAEs occurring more than thirty (30) days after discontinuation of the Product that are assessed by the Investigator as related to the Product should also be reported.

Follow-up Information: The IST Sponsor-Investigator will assist Seattle Genetics in investigating any SAE and will provide any follow-up information reasonably requested by Seattle Genetics.

Regulatory Reporting: Reporting an SAE to Seattle Genetics does not relieve the IST Sponsor-Investigator conducting the study of the responsibility for reporting it to the FDA, local regulatory authority, or IRB/IEC as required.

Western University 's Health Sciences Research Ethics Board will approve the protocol and any amendments and will be informed of any SAEs as per their policies. As per any trial, AEs and SAEs will be collected. SAEs will be reported in an expedited way to the UWO Ethics Committee if they change the protocol or Letter of Information as per UWO policy.

Data Safety Monitoring Board (DSMB)
The DSMB will be every 6 months and also at key time points (half the patients have been treated for 6 months) to review AEs, SAEs. They are independent of the trial and will recommend whether the trial can continue due to safety concerns.

All reporting of SAEs and AEs of interest will be done as per ICH guidance for clinical trials, Good Clinical Practice, recommendations of Health Canada and Seattle Genetics (sponsor of the drug).

12 SAMPLE SIZE

This study is a pilot and we have powered the sample size of 10 patients by expecting a large change in skin score. However, the effects on markers of fibrosis in serum and tissue specimens may help to determine if a larger trial should be a 'go' or 'no go'. We will consider data on the **first 10 patients enrolled** to determine if there is any potential benefit and consider safety but without any number of SAEs considering as a stopping rule as these patients are ill and SAEs related to rapidly progressive dcSSc are fairly common.

The sample size is based on science and convenience. For a mean change from end of study minus baseline mRSS of 8 and SD of 6, there should be 9 patients included in the study. In order to recruit at one site in a timely fashion, 10 active dcSSc patients who meet the inclusion criteria should be reasonable. The SD is based on baseline data from the imatinib trial where mean mRSS was 32 and SD at the beginning of the study was 8 and 7 at the end of the study and a more contemporary trial (tocilizumab) with a SD of 5.9 [24,31]. Dr. Pope has recruited for other single site studies such as with imatinib [24] as the London, Ontario scleroderma clinic is a large referral clinic.

With 10 patients we will be able to determine if the mRSS is decreased by at least 5 points on mRSS over 48 weeks.

Questionnaires will include:
SHAQ (Scleroderma HAQ-DI)
FACIT-Fatigue
EQ-5D
PASS (Patient Acceptable Symptom State)
Patient Global Assessment of Disease Activity
Physician (PI) Global Assessment of Disease Activity, severity and damage

13 ANALYSIS

This will be a modified intent to treat analysis, where any patient receiving at least one cycle of the study treatment will be analyzed, and for dropouts the last observation will be carried forward (LOCF).
Statistics will include the comparison of mRSS, FVC, DLCO, CRISS, SHAQ (HAQ-DI and VAS scales), inflammatory markers, and Health Transition Scores between baseline and follow-up visits by using paired tests. There will be analysis on skin biopsies pre and post Rx for CD30.
AEs and SAEs will be described.

14 FEASIBILITY

Dr. Pope is a clinical researcher (rheumatologist and epidemiologist) with an expertise in scleroderma including design of clinical trials. She runs a scleroderma program at St. Joseph's Hospital, affiliated with the University of Western Ontario, with a population that would allow rapid enrolment in this protocol and if needed, expanding enrolment from other large SSc clinics (Toronto and Montreal). She has successfully performed and published in other scleroderma trials, including designing and conducting as PI the largest trial of methotrexate in scleroderma. The CSRG (Canadian Scleroderma Research Group) is a group of 15 sites in Canada with dedicated rheumatologists who have a research interest in scleroderma. They have collectively more than 1300 scleroderma patients in a data set and have research staff familiar with clinical trials. The CSRG database will be used with selected (matched) historical controls to determine the potential benefit and safety of Brentuximab compared to the natural history of early dcSSc. We have used this methodology in past. All sites are experienced with skin biopsies and are co-authors on the CRISS.

References

1. Komócsi A, Vorobcsuk A, Faludi R et al. The impact of cardiopulmonary manifestations on the mortality of SSc: a systematic review and meta-analysis of observational studies. Rheumatology (Oxford). 2012; 5: 1027-1036.

2. Kowal‑Bielecka O, Veale DJ. DMARDs in systemic sclerosis: do they exist? In: Distler O, ed. Scleroderma‑modern aspects of pathogenesis, diagnosis and therapy. Uni‑MedVerlag AG: Bremen‑London‑Boston; 2009: 89-95.

3. Young A, Khanna D. Systemic sclerosis: a systematic review on therapeutic management from 2011 to 2014. Curr Opin Rheumatol. 2015;27(3):241-8.

4. Nevskaya T, Chandran S, Roos AM et al. Epidemiology of cancer in systemic sclerosis. Systematic review and meta-analysis of cancer incidence, predictors and mortality. Open Journal of Rheumatology and Ais not a cureutoimmune Dis, 2013; 3 (4): 231-245.

5. Shah A, Casciola-Rosen L and Rosen A. Cancer-Induced Autoimmunity in the Rheumatic Diseases. Arthritis and Rheumatoloy, 2015, 67(2): 317–326.

6. Andras C, Ponyi A, Constantin T et al. Dermatomyositis and polymyositis associated with malignancy: a 21-year retrospective study. J Rheumatol 2008;35:438–44.

7. Hasegawa M, Sato S, Sakai H et al. Systemic sclerosis revealing T-cell lymphoma. Dermatology 1999;198:75–8.

8. Juarez M, Marshall R, Denton C et al. Paraneoplastic scleroderma secondary to hairy cell leukaemia successfully treated with cladribine. Rheumatology (Oxford) 2008;47:1734–5.

9. Khanna D, Berrocal VJ, Giannini EH, Seibold JR, Merkel PA, Mayes MD, Baron M, et al. The American college of rheumatology provisional composite response index for clinical trials in early diffuse cutaneous systemic sclerosis. Arthritis Rheumatology 2016;68:299-311.

10. G. Valentini, A.J. Silman, D. Veale. Assessment of disease activity. Clin Exp Rheumatol 2003; 21 (Suppl. 29): S39-S41.

11. T.A. Medsger, S. Bombardieri, L. Czirjak et al. Assessment of disease severity and prognosis. Clin Exp Rheumatol 2003; 21 (Suppl. 29): S42-S46.

12. Oflazoglu E, Simpson EL, Takiguchi R, Grewal IS, Hanifin JM, Gerber H-P. CD30 expression on CD1a+ and CD8+ cells in atopic dermatitis and correlation with disease severity. Eur J Dermatol 2008;18:41-9.

13. Pope J, Baron M, Bellamy N. The variability of skin scores and clinical measurements in scleroderma. J Rheumatol. 1995; 22(7): 1271-1276.

14. Clements P, Lachenbruch P, Siebold J et al. Inter and intraobserver variability of total skin thickness score (modified Rodnan TSS) in systemic sclerosis. J Rheumatol. 1995; 22(7): 1281-5.

15. Clements PJ, Lachenbruch PA, Seibold JR et al. Skin thickness score in systemic sclerosis: an assessment of interobserver variability in 3 independent studies. J Rheumatol. 1993; 20(11): 1892-6.

16. Furst DE, Khanna D, Matucci-Cerinic M et al; OMERACT 7 Special Interest Group. Scleroderma—developing measures of response. J Rheumatol. 2005; 32(12): 2477-80.

17. Pope JE, Bellamy N. Outcome Measurement in Scleroderma Trials. Sem Arthritis Rheum. 1993; 23(1): 22-33.

18. Van den Hoogen F, Khanna D, Fransen J et al. Classification Criteria for Systemic Sclerosis: An ACR-EULAR Collaborative Initiative. Arthritis Rheum. 201; 65(11): 2737–2747.

19. LeRoy EC, Black C, Fleischmajer R, Jablonska S, Krieg T, Medsger TA, et al. Scleroderma (systemic sclerosis): classification, subsets and pathogenesis. J Rheumatol. 1988; 15(2): 202-5.

20. Sutherland MS, Sanderson RJ, Gordon KA, Andreyka J, Cerveny CG, Yu C, Lewis TS, Meyer DL, Zabinski RF, Doronina SO, Senter PD, Law C-L and Wahl AF (2006). Lysosomal trafficking and cysteine protease metabolism confer target-specific cytotoxicity by peptide-linked anti-CD30-auristatin conjugates. J Biol Chem 281: 10540-7.

21. Ong VH, Denton CP. Innovative therapies for systemic sclerosis. Curr Opin Rheumatol. 2010 May;22(3):264-72. doi: 10.1097/BOR.0b013e328337c3d6. Review.

22. Becker MO, Brückner C, Scherer HU, Wassermann N, et al. The monoclonal anti-CD25 antibody basiliximab for the treatment of progressive systemic sclerosis: an open-label study. Ann Rheum Dis. 2011 Jul;70(7):1340-1.

23. Oon S, Huq M, Godfrey T, Nikpour M. Systematic review, and meta-analysis of steroid-sparing effect, of biologic agents in randomized, placebo-controlled phase 3 trials for systemic lupus erythematosus. Semin Arthritis Rheum. 2018 Jan 6. pii: S0049-0172(17)30579-6. doi: 10.1016/j.semarthrit.2018.01.001

24. Pope J, McBain D, Petrlich L, Watson S, et al. Imatinib in active diffuse cutaneous systemic sclerosis: Results of a six-month, randomized, double-blind, placebo-controlled, proof-of-concept pilot study at a single center. Arthritis Rheum 2011 Nov;63(11):3547-51. doi: 10.1002/art.30549.

25. Gordon J, Martyanov V, Franks J, Bernstein E, et al. Belimumab for the treatment of early diffuse systemic sclerosis. Arthritis Rheumatol 2018 in press.

26. Nihtyanova S, Schreiber BE, Ong VH, Rosenberg D, et al Prediction of pulmonary complications and long-term survival in systemic sclerosis. Arthritis Rheumatol 2014 ;66:1625-35.

27. Boyang Z, Nevskaya T, Pope J, Baxter C, et al. Improvement in Skin Score after 2 Years in Early Diffuse Cutaneous Systemic Sclerosis (dcSSc) Patients is Associated with Improvement in Multiple Other Domains of Disease Measurement. J Rheumatol 2018 abstract from the Canadian Rheumatology Association Feb 20 meeting (Vancouver).

28. Van Laar J, Farge D, Sont JK, et al. Autologous hematopoietic stem cell transplantation vs intravenous pulse cyclophosphamide in diffuse cutaneous systemic sclerosis: a randomized clinical trial. JAMA 2014;311:2490-2498.

29. Sullivan KM, Goldmuntz EA, Keyes-Elstein L, McSweeney PA, et al. Myeloablative Autologous Stem-Cell Transplantation for Severe Scleroderma.N Engl J Med. 2018 Jan 4;378(1):35-47.

30. Fernández-Codina A, Walker KM, Pope JE. Real-Life Treatment Strategies for Systemic Sclerosis According to Experts. Arthritis Rheumatol. 2017; 69 (suppl 10).

31. Khanna D, Denton CP, Lin CJF, van Laar JM, et al. Safety and efficacy of subcutaneous tocilizumab in systemic sclerosis: results from the open-label period of a phase II randomised controlled trial (faSScinate). Ann Rheum Dis. 2018 Feb;77(2):212-220. doi: 10.1136/annrheumdis-2017-211682. Epub 2017 Oct 24. PMID: 29066464

32. Namas R, Tashkin DP, Furst DE, Wilhalme H, et al. Participants in the Scleroderma Lung Study I and members of the SclerodermaLung Study II Research Group. Efficacy of Mycophenolate Mofetil and Oral Cyclophosphamide on Skin Thickness: Post Hoc Analyses From Two Randomized Placebo-Controlled Trials. Arthritis Care Res (Hoboken). 2017 May 23. doi: 10.1002/acr.23282.

33. Gazi H, Pope J, Clements P, Medsger T, Martin R, Kahaleh B, et al. Outcome measurements in scleroderma. J Rheumatol. 2007 Mar;34(3):501-9.

34. Daoussis D, Melissaropoulos K, Sakellaropoulos G, Antonopoulos I, et al. A multicenter, open-label, comparative study of B-cell depletion therapy with Rituximab for systemicsclerosis-associated interstitial lung disease. Semin Arthritis Rheum. 2017 Apr;46(5):625-631.

35. Brentuximab vedotin product monograph from FDA. https://www.accessdata.fda.gov/drugsatfda_docs/label/2014/125388_S056S078lbl.pdf Searched Feb 20, 2018.

36. Ramiro S, Sepriano A, Chatzidionysiou K, Nam JL, et al. Safety of synthetic and biological DMARDs: a systematic literature review informing the 2016 update of the EULAR recommendations for management of rheumatoid arthritis. Ann Rheum Dis. 2017 Jun;76(6):1101-1136

37. Kivitz A, Olech E, Borofsky M, Zazueta B, Navarro-Sarabia F, Radominski S, et al. The Safety and Efficacy Of Tocilizumab Subcutaneous In Combination With Traditional Dmards In

Patients With Moderate To Severe Rheumatoid Arthritis Up To 48 Weeks (BREVACTA). Arthritis Rheum 2013;65 Suppl 10 :1428 DOI: 10.1002/art.2013.65.issue-s10.

Appendix
Abstracts

Reference 27 abstract

Improvement in Skin Score after 2 Years in Early Diffuse Cutaneous Systemic Sclerosis (dcSSc) Patients is Associated with Improvement in Multiple Other Domains of Disease Measurement

Boyang Zheng (McGill University Health Center, Montreal); Tatiana Nevskaya (Rheumatology Research, St. Joseph's Health Care, London); Janet Pope (Western University, Department of Medicine, Division of Rheumatology, London); Carl Baxter (MSD Ltd, Hoddesdon); Dena Ramey (Merck & Co., Inc., , Kenilworth); Murray Baron (McGill University, Jewish General Hospital, Montreal); Canadian Scleroderma Research Group (CSRG) (Montreal)

Objectives: Drug trials in dcSSc often use the change in modified Rodnan skin score (mRSS) as a primary outcome. The goal of this study was to determine if improvement in skin over time is associated with improvement in other domains in early dcSSc patients.

Methods: dcSSc patients with ≤5 years disease duration followed over 2 years were identified from the Canadian Scleroderma Research Group registry. Changes in the degree of organ involvement were assessed using the Medsger Disease Severity Score (DSS) for each of the 9 systems as both continuous and dichotomous variables. A ≥1 point decrease was considered a clinical improvement. Pulmonary function tests, patient and physician reported global measures, Health Assessment Questionnaire (HAQ) and SF-36 physical component score (PCS) were also assessed. Bivariate, ANOVA and linear correlation (Pearson) analyses were performed. **Results:** Of the 128 patients with 2 year follow up, 50% demonstrated skin improvement (mRSS decrease of ≥5 points and/or a ≥25% reduction). Over two years, the average mRSS decreased from 22.6 to 18.1 for the entire cohort (p=0.0001). Physician global assessments of disease severity, activity and damage improved more in patients whose skin score improved (p<0.003). Improving mRSS was correlated with improving DSS for the following organ systems: lung, kidney, gastrointestinal, peripheral vascular, and the sum of all DSS scores without skin DSS. Compared to non-improvers, a greater proportion of skin-improvers had clinical lung improvement (39.4% vs 17.2%, p=0.006). Improvement in Forced Vital Capacity % predicted (FVC%) also correlated with skin improvement (r=0.33, p=0.004). FVC% was stable in skin- improvers (0.04% increase) while declining by 6.5% in non-improvers, p=0.026. A higher proportion of skin-improvers also had improvement in joint/tendon involvement (50% vs 21.2%, p=0.017) and improvement in any visceral organ involvement (renal, cardiac, pulmonary or gastrointestinal) (60.3% vs 27.3%, p=0.031). Skin-improvers had significantly improved HAQ scores compared to non-improvers (-0.19 point decrease vs. 0.18 point increase, p=0.001). Similarly, SF-36 PCS improved by 3.1 points in skin-improvers and worsened by 1.6 points in non-improvers, p=0.005.

Conclusion: Over two years, improving skin scores in dcSSc patients were associated with an improvement in lung disease, joint/tendon involvement, global physician assessments, HAQ and SF-36 PCS. Skin-improvers had more overall visceral organ improvement. Our findings support the hypothesis that improvement in the severity of skin disease as a primary outcome in drug trials may be a surrogate for improvement in organ involvement and several other disease measurement domains.

Reference 30 abstract

Treatment algorithms for systemic sclerosis according to experts

Authors: Fernández-Codina, Andreu[1,2]; Walker, Kyle M[3]; Pope, Janet E[1*] on behalf of the Scleroderma Algorithm Group[#].

Introduction. Treatment for many aspects of systemic sclerosis (SSc) lacks agreement.
Objectives. To generate SSc treatment algorithms endorsed by high percentage of SSc experts.
Methods. Experts from the Scleroderma Clinical Trials Consortium and the Canadian Scleroderma Research group (N=170) were asked whether they agreed with SSc algorithms (from 2012). A further 2 consensus rounds refined agreement; 62 (36%), 54 and 48 experts completed surveys.
Results. For scleroderma renal crisis (SRC), 82% of the experts agreed (1st line ACEi, 2nd and 3rd adding: CCB or ARB). Pulmonary arterial hypertension (PAH) had 81% agreement. For mild PAH, PDE5i, then endothelin receptor antagonists plus PDE5i, then prostanoids; while for severe PAH prostanoids were first-line. Raynauds' phenomenon (RP) had 78% of agreement [mild (1st CCB, 2nd adding PDE5i, 3rd ARB or switching to another CCB, 4th prostanoids), severe (1st CCB, 2nd adding PDE5i, 3rd ERA, 4th prostanoids)]. Digital ulcer (DU) treatment had 69% agreement (1st CCB, 2nd PDE5i). Interstitial lung disease (ILD) had 65% agreement including induction (Mycophenolate mofetil (MMF) then intravenous cyclophosphamide then rituximab) and maintenance (1st line MMF). Skin involvement had 71% agreement. For a modified Rodnan skin score (mRSS) of 24 1st MTX, 2nd MMF; and for mRSS 32 1st MMF, 2nd MTX, 3rd intravenous cyclophosphamide (CYP), 4th hematopoietic stem cell transplantation. For inflammatory arthritis 79% agreed with 1st MTX, 2nd low dose glucocorticoids, 3rd hydroxychloroquine, 4th rituximab or tocilizumab. Cardiac and gastrointestinal algorithms had $\geq$75% agreement.
Conclusions. Total agreement for SSc algorithms was considerable. These SSc algorithms may guide treatment.

Slide presentation to Health Canada
