# Supplementary material for: Brentuximab vedotin for skin involvement in refractory diffuse cutaneous systemic sclerosis, an open-label trial
Source: Rheumatology (Oxford). 2024 Apr 23;64(3):1476–81. doi: 10.1093/rheumatology/keae235 (PMC11879290; doi:10.1093/rheumatology/keae235)
Supplement: keae235_Supplementary_Data [file keae235_supplementary_data.zip › keae235_Supplementary_Data/rhe-24-0405-File009.docx]

Inclusion criteria:

1. Patients with scleroderma, aged 18 years or older.
2. Subjects met the 2013 ACR/EULAR classification criteria.
3. Early dcSSc (within 5 years of first non-RP symptom) or active dcSSc as determined by worsening mRSS, presence of tendon friction rubs, and/or elevated inflammatory markers thought to be due to active dcSSc and not related to other issues such as infection.
4. mRSS ≥15.
5. Able to give informed consent.
6. Negative TB skin test at screening, or treatment with isoniazid for 6 months or other standardized latent tuberculosis (TB) treatment in the past.

Exclusion Criteria:

1. Poor pulmonary function (FVC <40% and/or DLCO <30%).
2. Pregnancy, breast feeding, or childbearing potential without practicing reliable contraception (and partners for men in the study).
3. Clinically significant pulmonary hypertension requiring drug therapy.
4. Clinically significant cardiac disease.
5. Chronic or ongoing active infectious disease requiring systemic treatment including
   1. Seropositivity for human immunodeficiency virus at study entry.
   2. Active TB infection.
   3. Active viral infection with viral replication of hepatitis B or C virus at study entry.
6. Significant concurrent, uncontrolled medical condition including, but not limited to renal, hepatic, pancreatic, haematological, gastrointestinal, endocrine, pulmonary, neurological, cerebral or psychiatric disease, and/or cancer.
7. Peripheral neuropathy at screening Grade 2 or higher.
8. Patients known or suspected of not being able to comply with a study protocol (e.g. due to alcoholism, drug dependency, or severe psychological disorder)
9. Any of the following laboratory abnormalities at screening:
   -Absolute neutrophils count <2.0 x 109/L
   -Hemoglobin <85 g/L
   -Platelet count <100 x 109/L
   -AST/SGOT >2-fold upper normal level
   -ALT/SGPT >2-fold upper normal level
10. Participation in another clinical trial within six weeks before randomization in this study
11. Use of rituximab within the previous 4 months.
12. Immunization with a live/attenuated vaccine less than 4 weeks prior to the baseline visit.
13. Previous use of brentuximab vedotin.
14. Current or history of progressive multifocal leukoencephalopathy.

Serum cytokine exams

Whole blood was obtained by venipuncture at baseline and every 3 weeks until week 48. It was placed in serum separator tubes and spun for 15 minutes. Serum was collected and frozen at -80ºC. Inflammatory markers in the serum were explored using the Human Soluble Cytokine Receptor 14-Plex Discovery Assay® Array (Eve Technologies, Calgary, AB, Canada) testing for CD30 and other pathways. The assay was used as per the manufacturer’s instructions.

Pathology exams

The skin biopsies were performed under local anesthesia with lidocaine using a 3mm punch, obtaining two samples each time at weeks 0, 24, and 48. Samples were immersed in formalin for storage. Fixed tissue was run through a tissue processor to dehydrate and imbue the tissue with paraffin wax. 5µm sections were collected on slides using a microtome. Immunostaining was done using a Dako Omnis system (Agilent, Santa Clara, CA, USA). The skin biopsies were stained with Hematoxylin (Harris hematoxylin. Leica, Deer Park, IL, USA) & Eosin (Eosin Y. Fischer scientific. Waltham, MA, USA), Lillie’s trychrome (Fischer scientific. Waltham, MA, USA), an anti-CD4 mouse monoclonal antibody (CD4 Autostainer Link 48 clone 4B12. Agilent, Santa Clara, CA, USA), and an anti-CD30 mouse monoclonal antibody (CD30 Autostainer Link 48 clone Ber-H2. Agilent, Santa Clara, CA, USA). The assays were used as per the manufacturer’s instructions.

Supplementary Table 1. Laboratory data for patients treated with brentuximab vedotin

| **Mean (SD)** | **Week 0** | **Week 48** | **Mean difference** |
| --- | --- | --- | --- |
| **Hemoglobin mg/dL** | 112.9 (12.6) | 120.2 (13.8) | -4.1 (95% CI -12, 3.4); p=0.2 |
| **Leukocytes cell/mm3** | 6.4 (1.6) | 6.4 (2.3) | -0.2 (95% CI -1.6, 1.2); p=0.8 |
| **Neutrophils cell/mm3** | 4.6 (1.4) | 4.5 (2) | -0.1 (95% CI -1.3, 1); p=0.8 |
| **Platelets cell/mm3** | 282 (135.6) | 251.1 (70.5) | -9.8 (95% CI -29, 9.6); p=0.3 |
| **ESR mm/h** | 21.8 (12.7) | 15.5 (12.9) | 4.5 (95% CI -3.8, 13); p=0.2 |
| **CRP mg/dL** | 5.2 (5.3) | 3.3 (2.2) | 0.7 (95% CI -3.2, 4.6); p=0.7 |
| **ALT IU/L** | 10.7 (5.5) | 17.4 (11) | -6.2 (95% CI -13, 0.9); p=0.079 |
| **Glucose mmol/L** | 5.8 (1.2) | 4.9 (0.2) | 0.9 (95% CI -0.2, 2.1); p=0.091 |

SD=standard deviation; ESR=Erythrocyte sedimentation rate; CRP=C reactive protein; ALT=alanine aminotransferase; CI=confidence interval

Supplementary Table 2. Patient/physician reported outcomes for patients treated with brentuximab vedotin

| **Item Mean (SD)** | **Week 0** | **Week 48** | **Mean difference** |
| --- | --- | --- | --- |
| PGA N=8 | 7 (2) | 4.4 (2.7) | 2.4 (95% CI 0.8, 4); p=0.008 |
| HAQ-DI N=8 | 1.708 (0.612) | 1.422 (0.858) | -0.31 (95% CI -0.14, 0.76); p=0.14 |
| FACIT-F N=8 | 21.8 (12.4) | 28 (12.6) | -7.5 (95% CI -17, 2.3); p=0.11 |
| MDGA severity N=8 | 5.6 (1.9) | 2.1 (1.1) | 2.7 (95% CI 1.1, 4.4); p=0.007 |
| MDGA activity N=8 | 5.6 (1.9) | 2.4 (1.2) | 3 (95% CI 1.5, 4.5); p=0.003 |
| MDGA damage N=8 | 5 (1.5) | 3.6 (1.1) | 1.4 (95% CI 0.12, 2.6); p=0.036 |
| VAS-pain N=9 | 41.6 (34.6) | 42.2 (30.4) | -0.7 (95% CI -27, 26); p>0.6 |
| VAS-intestinal N=9 | 26.7 (31.5) | 32 (32.4) | -5.3 (95% CI -29, 19); p=0.6 |
| VAS-dyspnea N=9 | 19.1 (33.1) | 14.9 (29.5) | 4.2 (95% CI -0.6, 9); p=0.078 |
| VAS-Raynaud’s N=9 | 33.7 (28.2) | 33.9 (27.2) | -0.2 (95% CI -13, 13); p>0.9 |
| VAS-digital ulcer N=9 | 34.3 (37.2) | 25.1 (35.4) | 9.2 (95% CI -15, 34); p=0.4 |
| VAS-severity N=9 | 66.3 (24.3) | 47.2 (27.9) | 19 (95% CI 5.1, 33); p=0.014 |
| **Status N (%)** | **Week 12**  **N=9** | **Week 48**  **N=9** | **Mean difference** |
| PASS1 | 6 (67) | 7 (78) | -11 (95% CI 57, 35); p=0.6 |
| PASS2 | 3 (33) | 3 (33) | 0 (95% CI -54, 54); p=>0.9 |
| PASS3 | 4 (44) | 7 (78) | -33 (95% CI -72, 5.1); p=0.081 |
|  |  |  |  |

SD=standard deviation; PGA=patient general assessment (0-10); HAQ-DI=health assessment questionnaire-disability index (0-3); FACIT-F=functional assessment of chronic illness therapy-fatigue (0-52); MDGA=physician general assessment (0-10); VAS-pain: visual analogue scale for pain in the previous week (0-100); VAS-intestinal: visual analogue scale for intestinal problems interference with daily activities in the previous week (0-100); VAS-dyspnea: visual analogue scale for dyspnea interference with daily activities in the previous week (0-100); VAS-Raynaud´s: visual analogue scale for Raynaud´s phenomenon interference with daily activities in the previous week (0-100); VAS-digital ulcer: visual analogue scale for digital ulcer interference with daily activities in the previous week (0-100); VAS-severity: visual analogue scale for overall severity of disease considering pain, discomfort, limitations with daily activities (0-100); CI=confidence interval; PASS=patient acceptable symptom state

Supplementary Table 3. Grade of skin fibrosis over time in patients treated with brentuximab vedotin

| **Fibrosis grade** | **Week 0**  **N=10** | **Week 24**  **N=8** | **Week 48**  **N=7** |
| --- | --- | --- | --- |
| None  N (%) | 0 (0) | 0 (0) | 0 (0) |
| Mild | 0 (0) | 0 (0) | 0 (0) |
| Moderate | 7 (70) | 6 (75) | 5 (71) |
| Severe | 3 (30) | 2 (25) | 2 (29) |
|  |  |  |  |

No statistically significant changes (Fisher`s exact test)

Supplementary Figure 1. Evolution of the skin fibrosis in a patient treated with brentuximab vedotin


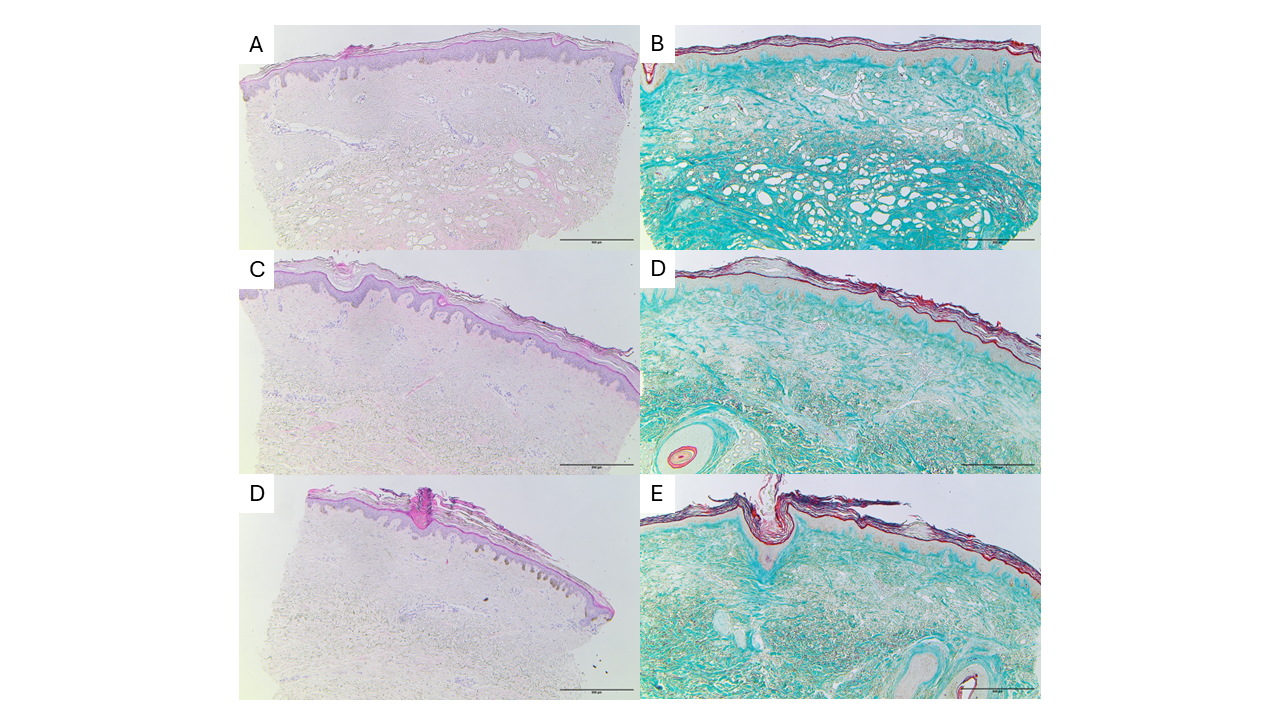


Optic microscope 72x. Hematoxylin & Eosin (panels a, c, e). Lillie’s trichrome (panels b, d, f). The density of the sclerosed collagen bundles in the dermis did not show changes for the same patient from week 0 (panels a and b) to week 24 (panels c and d) and week 48 (panels e and f)
